# Profibrogenic role of IL-15 through IL-15 receptor alpha-mediated trans-presentation in the carbon tetrachloride-induced liver fibrosis model

**DOI:** 10.3389/fimmu.2024.1404891

**Published:** 2024-06-11

**Authors:** Maryse Cloutier, Bhavesh Variya, Sara Ali Akbari, Fjolla Rexhepi, Subburaj Ilangumaran, Sheela Ramanathan

**Affiliations:** Department of Immunology and Cell Biology, Faculty of Medicine and Health Sciences, Université de Sherbrooke, Sherbrooke, QC, Canada

**Keywords:** IL-15, liver fibrosis, IL-15Rα, carbon tetra chloride (CCl) 4, pre-clinical study

## Abstract

**Background:**

Inflammatory cytokines play key pathogenic roles in liver fibrosis. IL-15 is a proinflammatory cytokine produced by myeloid cells. IL-15 promotes pathogenesis of several chronic inflammatory diseases. However, increased liver fibrosis has been reported in mice lacking IL-15 receptor alpha chain (IL-15Rα), suggesting an anti-fibrogenic role for IL-15. As myeloid cells are key players in liver fibrosis and IL-15 signaling can occur independently of IL-15Rα, we investigated the requirement of IL-15 and IL-15Rα in liver fibrosis.

**Methods:**

We induced liver fibrosis in *Il15^–/–^*, *Il15ra^–/–^* and wildtype C57BL/6 mice by the administration of carbon tetrachloride (CCl_4_). Liver fibrosis was evaluated by Sirius red and Mason’s trichrome staining and α-smooth muscle acting immunostaining of myofibroblasts. Gene expression of collagens, matrix modifying enzymes, cytokines and chemokines was quantified by RT-qPCR. The phenotype and the numbers of intrahepatic lymphoid and myeloid cell subsets were evaluated by flow cytometry.

**Results:**

Both *Il15^–/–^* and *Il15ra^–/–^* mice developed markedly reduced liver fibrosis compared to wildtype control mice, as revealed by reduced collagen deposition and myofibroblast content. *Il15ra^–/–^* mice showed further reduction in collagen deposition compared to *Il15^–/–^* mice. However, *Col1a1* and *Col1a3* genes were similarly induced in the fibrotic livers of wildtype, *Il15^–/–^* and *Il15ra^–/–^* mice, although notable variations were observed in the expression of matrix remodeling enzymes and chemokines. As expected, *Il15^–/–^* and *Il15ra^–/–^* mice showed markedly reduced numbers of NK cells compared to wildtype mice. They also showed markedly less staining of CD45^+^ immune cells and CD68^+^ macrophages, and significantly reduced inflammatory cell infiltration into the liver, with fewer pro-inflammatory and anti-inflammatory monocyte subsets compared to wildtype mice.

**Conclusion:**

Our findings indicate that IL-15 exerts its profibrogenic role in the liver by promoting macrophage activation and that this requires trans-presentation of IL-15 by IL-15Rα.

## Introduction

Liver fibrosis is a chronic inflammatory disease characterized by the accumulation of extra-cellular matrix proteins due to the failure to resolve inflammation and to repair tissue damage (1, 2). Hepatitis viruses, bacterial pathogens and alcohol abuse are the major etiologic factors in liver fibrosis. With the global raise in obesity and metabolic syndrome, the consequent non-alcoholic fatty liver disease has emerged as another major contributor to liver fibrosis (3). Unchecked liver fibrosis can progress toward cirrhosis, wherein extensive replacement of the liver parenchyma by fibrous tissue compromises vital liver functions. Cirrhosis is an important cause of global morbidity and mortality (4). Hepatocyte proliferation within the inflammatory setting of liver fibrosis and cirrhosis promotes the development of hepatocellular carcinoma, a widely prevalent cancer and an important cause of cancer related death (5). Whereas, advanced cirrhosis is irreversible, resolution of liver fibrosis can be achieved through the removal of the causative factors and by inhibiting the pathogenic signaling pathways of fibrogenesis (6, 7).

During liver fibrosis, inflammatory stimuli arising from diverse sources such as pathogen-associated molecules and danger-associated molecules released from stressed and damaged hepatocytes initiate the hepatic fibrogenic response, which is amplified by inflammatory mediators released by resident and recruited immune cells to contain tissue damage and facilitate repair (2, 8, 9). Activation of liver-resident Kupffer cells (KC) and hepatic stellate cells (HSC) initiate the fibrogenic process by producing inflammatory and fibrogenic cytokines such as TNFα, IL-6, IL-1β and TGFβ, as well as growth factors (7, 10). These soluble mediators promote activation and proliferation of HSCs, which produce the extracellular matrix components to facilitate tissue repair. Increased production and dysregulated signaling of inflammatory and fibrogenic cytokines are implicated in the excess production and accumulation of abnormal matrix components during liver fibrosis (6, 7, 11).

The liver harbors a wide variety of lymphoid cells including NK cells, NKT cells and CD8^+^ T lymphocytes, which are reported to play antifibrotic, profibrotic or both roles, depending on the context (9, 12, 13). These cells rely on IL-15 for their homeostasis (14–16). IL-15 is a member of the IL-2 family of cytokines and signals via the trimeric IL-15 receptor (IL-15R) complex, which consists of the ligand-specific IL-15Rα chain, the β subunit (also shared by IL-2R, hence called IL-2/15Rβ), and the common γ (γ_c_) chain (17, 18). IL-15 is induced in response to inflammatory stimuli and macrophages are major producers although epithelial cells including hepatocytes can also provide IL-15 (19, 20). IL-15 protein expression occurs via a unique biosynthetic pathway. IL-15 associates with IL-15Rα during biosynthesis, and this complex is ‘*trans*-presented’ to cells that express IL-15Rβγ_c_ receptor (17, 18, 21). IL-15 trans-presentation is crucial for the homeostasis of T cells and NK cells (21). In biological fluids IL-15 exists predominantly as soluble biologically active heterodimeric IL-15/IL-15Rα (22–24). In the liver, IL-15 and its receptors are expressed in hepatocytes, HSCs, and macrophages (20, 25, 26). We and others have shown that trans-presentation of IL-15 by IL-15Rα on hepatocytes, HSCs and macrophages is required for the maintenance of hepatic NK, NKT and CD8^+^ T cells (16, 20, 26).

Development of non-alcoholic fatty liver disease was attenuated in mice deficient for IL-15 or IL-15Rα (16, 27, 28), supporting a pro-inflammatory role of IL-15 in the liver. However, the current literature on the role of IL-15 in liver fibrosis are contradictory. In patients co-infected with HCV and HIV, IL-15 expression is correlated to HSC activation and accelerated liver fibrosis (29). Similarly, a minor SNP at the 3’-untranslated region of *IL15* gene (rs10833 AA instead of GG/GA) increases liver fibrosis risk in HCV/HIV co-infected patients by 2.3-fold via unknown mechanisms (30). IL-15 produced by activated HSCs was shown to promote liver fibrosis by increasing the survival and pathogenic potential of neutrophils (31). In contrast to the above reports indicating a pro-fibrogenic role for IL-15, increased liver fibrosis has been reported in IL-15Rα deficient mice (32). This was attributed to a direct inhibitory effect of IL-15Rα signaling on the fibrogenic response of HSCs. However, we have shown that IL-15 mediated bacterial clearance and autoinflammatory pathologies can occur in the absence of IL-15Rα (33, 34). Moreover, the susceptibility of IL-15 deficient mice to liver fibrosis has not yet been studied. In the present work, we used both *Il15^–/–^* and *Il15ra^–/–^* mice to understand the role of IL-15 in chemical induced liver fibrosis and the requirement for IL-15Rα in mediating IL-15 signaling during liver fibrosis. Our findings reveal that IL-15 exerts a profibrogenic role in liver fibrosis induced by carbon tetrachloride (CCl_4_) and its pathogenic effects are largely dependent on IL-15Rα-mediated trans-presentation of IL-15.

## Materials and methods

### Animals

Wildtype, *Il15^–/–^* and *Il15ra^–/–^* mice in the C57Bl/6 background generated in our colony have previously been described (27, 34). *Il15^–/–^* and *Il15ra^–/–^* mice were back-crossed to C57BL/6J mice (Charles River, Canada) every four generations, and the three genotypes used in this study were established from these crosses. Analyses of gut microbiota of the three genotypes showed minimal differences (35). Mice were bred and housed in ventilated cages in the same housing unit throughout the experiment. The experimental protocols were approved by Animals Ethics committee of the Faculty of Medicine and Health Sciences, Université de Sherbrooke (2020-2732).

### Induction of liver fibrosis

Liver fibrosis was induced in 8-10 week-old mice by intraperitoneal (i.p.) injection of CCl_4_ as previously described (36). Male mice were used for fibrosis induction as female sex hormones reduced production of inflammatory cytokine in the liver (37). Briefly, CCl_4_ (Sigma-Aldrich, Oakville, ON) diluted in corn oil (1:3) was injected via i.p. route (0.5 mL CCl_4_ per g of body weight) twice a week for five weeks. Three to four days after the last treatment, mice were sacrificed, blood collected by cardiac puncture and liver and spleen tissues resected. Serum was separated and kept frozen at -80°C. Liver pieces were snap frozen and stored at -80°C for gene expression studies. For histopathology analyzes, tissue sections from the different liver lobes were fixed for 12-16 hours in buffered 4% paraformaldehyde solution and embedded in paraffin.

### Serum Alanine Aminotransferase assay

Quantitative determination of serum Alanine Aminotransferase (ALT) was performed using a kinetic assay according to the manufacturer’s instruction (Pointe Scientific, MI, USA).

### Histology and immunofluorescence

Formalin-fixed paraffin-embedded tissue sections of 4 μm thickness were deparaffinized, rehydrated, and stained with hematoxylin and eosin (H&E), Sirius red/Fast green in Picric Acid (Fast green FCF, EMD Millipore Corporation, MA, USA) or Masson’s trichrome stain following standard procedures (36). Digital images of the stained sections were acquired using a Nanozoomer Slide Scanner (Hamamatsu Photonics, Japan) and analyzed by the Nanozoomer Digital Pathology software NDPview 2.7.52 (Hamamatsu Photonics). Sirius red staining positive areas were quantified using the NIH ImageJ software (version 1.53n). For immunohistochemistry, rehydrated liver tissue sections were immersed in Tris-EDTA Buffer (10mM Tris Base, 1mM EDTA Solution, 0.05% Tween 20, pH 9.0) and maintained at 90°C, 20 min in a steamer for antigen retrieval. Sections were then blocked with 5% BSA in Tris-buffered saline (TBS) containing 20% Tween-20 (TBS-T) followed by overnight incubation with primary antibody at 4°C. After washing, tissue sections were incubated with appropriate secondary antibody for 2h, washed and nuclei were stained using Hoechst 33342 DNA staining dye (Thermo Fisher Scientific, Canada). After washing, tissue sections were mounted with a coverslip. The list of antibodies used are listed in **Supplementary Table 1**. Images were acquired in Nanozoomer and were analyzed using NDP-View2 and ImageJ software (Hamamatsu Photonics, Japan).

### Gene expression analyzes

Quantitative RT-PCR was carried out as described before (36). Briefly, Total RNA from frozen tissues was extracted using QIAzol Lysis Reagent (Qiagen, Toronto, Ontario, Canada). cDNA was synthetized from 1μg of purified RNA using QuantiTect^®^ reverse transcription kit (Qiagen, Toronto, Ontario, Canada). Quantitative RT-PCR amplification reactions was carried out in QuantStudio 3 Real-Time PCR System (Thermo Fisher Scientific, Canada) using SYBR Green Supermix (Bio-Rad, Mississauga, Ontario, Canada). The expression of indicated genes was measured using primers listed in **Supplementary Table S2**. Gene expression levels between samples were normalized based on the Cycle threshold (Ct) values compared to housekeeping gene *36B4* (*Rplp0*). Statistical analyzes were performed using GraphPad Prism 9 software (San Diego, CA). The values are presented as mean ± standard error of the mean (SEM). The statistical significance (*p* value) was calculated as indicated in figure legends.

### Isolation of IHLs and flow cytometric analyzes

Intrahepatic lymphocytes were isolated as described (38). At sacrifice, liver tissues were collected, rinsed with Krebs–Ringer–Buffer (KRB; 154 mM NaCl, 5.6 mM KCl, 5.5 mM Glucose, 20.1 mM HEPES, 25 mM NaHCO3, pH 7.4) and digested in pre-warmed (37°C) KRB supplemented with 2 mM CaCl_2_, 2 mM MgCl2, 300 CDU (casein digestion units)/mL Collagenase IV (Worthington) and 150 U/mL DNase I (Sigma) using the GentleMACS™ Dissociator (Miltenyi Biotec, Cambridge, MA, US) following the manufacturer’s instructions. The homogenized liver samples were incubated for 30 min at 37°C under gentle agitation, followed by a second round of digestion using the GentleMACS™ Dissociator. The digested liver tissues were passed through a 70 μm mesh size cell strainer and rinsed with cold PEB buffer (0.5% bovine serum albumin, 2 mM EDTA in Phosphate buffered saline). Samples were centrifuged at 50*g* for 5 min, at 4°C to eliminate contaminating hepatocytes. The supernatant was centrifuged at 300*g* for 10 min at 4°C to collect the leukocytes. Cells were further purified using a Percoll gradient as described (38), rinsed with PEB buffer and used for flow cytometry. Data were acquired on Cytoflex using CytExpert software (BeckMan Coulter, Indianapolis, IN) and analyzed using the FlowJo software (TreeStar Inc, Ashland, OR). Antibody panels used for identifying T cells and myeloid subsets are listed in **Supplementary Table 3**.

## Results

### IL-15 promotes liver fibrosis through IL-15Rα

**Figure 1:**
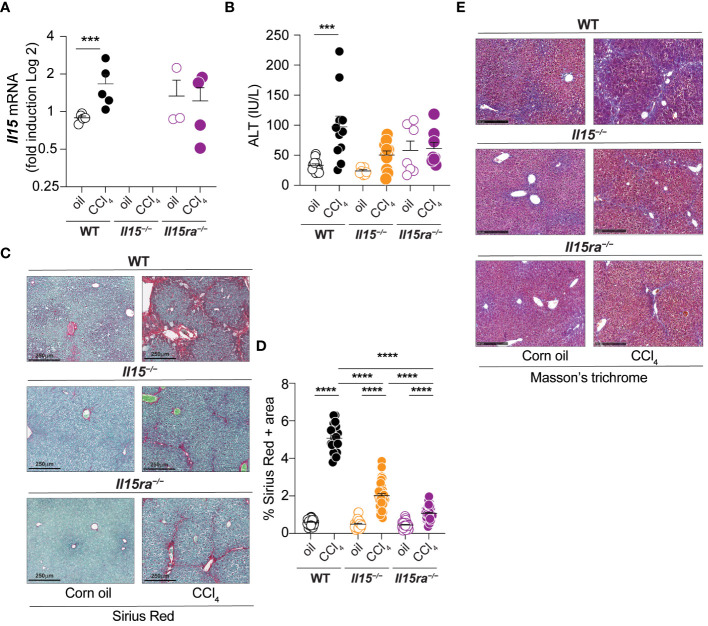
IL-15 or IL-15Rα deficiency reduces CCl_4_-induced liver fibrosis. **(A)** *Il15* gene expression was determined by RT-qPCR in the livers of wildtype (WT), *Il15^–/–^* and *Il15ra^–/–^* mice treated with vehicle (oil) or CCL_4_. **(B)** Serum ALT levels in WT, *Il15^–/–^* and *Il15ra^–/–^* mice following vehicle or CCl_4_ treatment at the endpoint. Values from individual mice are shown. **(C)** Sirius Red/Fast green staining of WT, *Il15^–/–^* and *Il15ra^–/–^* liver tissues from oil- or CCl_4_-treated mice. Data shown are representative of at least 8 mice per group from 3 to 4 separate experiments. **(D)** Quantification of Sirius Red stained area was performed on seven to ten were randomly selected fields in the different regions of liver sections from 3 to 4 mice per group. **(E)** Masson’s trichrome staining of WT, *Il15^–/–^* and *Il15ra^–/–^* liver tissues from oil and CCl_4_-treated mice. Data shown are representative of 4-5 mice per group from three independent experiments. **(A, B, D)** Mean + standard error of the mean (SEM). Statistical significance was calculated using Mann-Whitney test **(A)** or ordinary one-way ANOVA with Tukey’s post-hoc test: ***p<0.001; ****p<0.0001.

To understand the role of IL-15 signaling in hepatic fibrogenic response, male wildtype (WT), *Il15^–/–^* and *Il15ra^–/–^* mice were treated with CCl_4_ for 5 weeks to induce of liver fibrosis. We first examined the expression of the *Il15* gene in CCl_4_-treated livers. Wildtype mice showed a marked increase *Il15* gene expression following CCl_4_ treatment, whereas such an upregulation was not evident in the livers of *Il15ra^–/–^* mice (**Figure 1A**). Serum alanine transferase (ALT) levels were higher in CCl_4_-treated wildtype mice when compared to vehicle-treated controls (**Figure 1B**). In contrast, no difference in ALT levels were observed between control and CCl_4_-treated *Il15^–/–^* and *Il15ra^–/–^* mice, suggesting that CCl_4_-induced liver damage is amplified by IL-15 signaling via IL-15Rα. Sirius red staining of liver sections for collagen fibers revealed extensive liver fibrosis in CCl_4_-treated wildtype mice when compared to the vehicle-treated controls with marked portal to portal bridging fibrosis (**Figure 1C**). Liver sections of CCl_4_-treated *Il15^–/–^* and *Il15ra^–/–^* mice showed significantly reduced collagen deposition compared to CCl_4_-treated wildtype mice, with collagen deposition in portal areas and occasional portal to portal bridging (**Figure 1C**). Quantification of the Sirius red-staining area showed significantly less fibrosis in CCl_4_-treated *Il15ra^–/–^* mice than in *Il15^–/–^* mice (**Figure 1D**). Masson’s trichrome staining corroborated with the Sirius red staining pattern and intensity (**Figure 1E**). These results indicate that IL-15 plays a profibrogenic role in liver fibrosis pathogenesis, and this is mediated by IL-15Rα-dependent IL-15 signaling.

### Variable induction of hepatic fibrogenic genes in the absence of IL-15 or IL-15Rα

**Figure 2:**
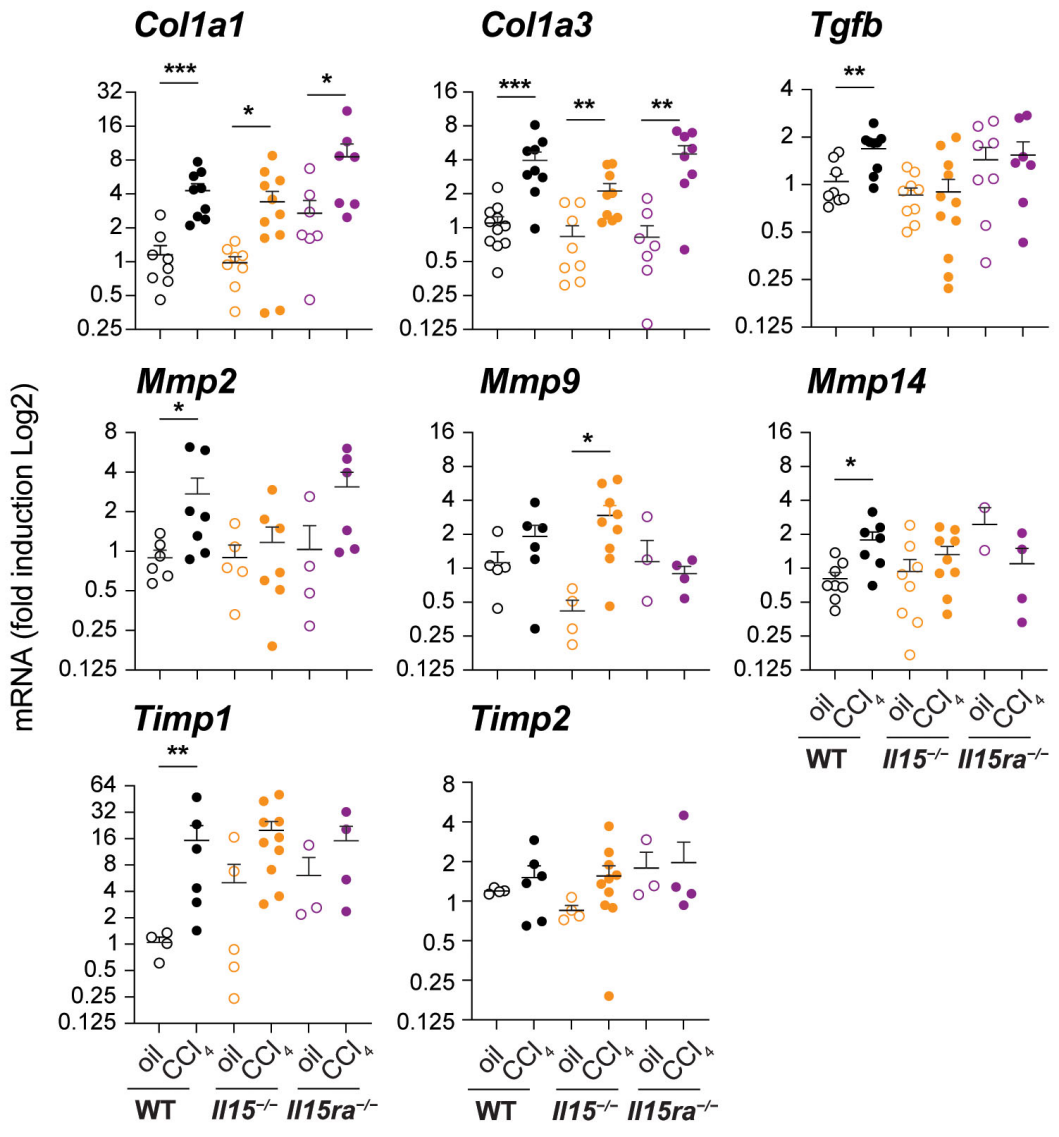
Impact of IL15 or IL15Rα deficiency on the expression of fibrogenic genes. Indicated genes associated with hepatic fibrogenesis were assessed in the livers of oil- or CCl_4_- treated mice from the three genotypes. Mean + SEM. Statistical significance was calculated using Mann-Whitney’s test: *p<0.5; **p<0.1; ***p<0.001.

Next, we examined the expression of genes associated with hepatic fibrogenic response in the livers of CCl_4_-treated and wildtype, *Il15^–/–^* and *Il15ra^–/–^* mice. CCl_4_-treated wildtype mice livers showed increased expression of *Col1a1* and *Col1a3* genes coding for collagens (**Figure 2**), corroborating with increased collagen deposition observed by Sirius Red and Masson’s trichrome staining (**Figures 1C–E**). Livers of CCl_4_-treated *Il15^–/–^* and *Il15ra^–/–^* mice also showed upregulation of *Col1a1* and *Col1a3* genes that was comparable to wildtype mice (**Figure 2**), although collagen deposition in IL-15 or IL-15Rα deficient mice was not increased to the extent observed in wildtype mice (**Figures 1C–E**). Notably, the induction of the pro-fibrogenic cytokine TGFβ (*Tgfb*) was increased in CCl_4_-treated wildtype livers compared to the corresponding control livers, but not in the livers of *Il15^–/–^* and *Il15ra^–/–^* mice (**Figure 2**). Induction of the antifibrotic matrix metalloproteinase 2 (*Mmp2*) (39), was increased in wildtype mice but not in *Il15^–/–^* mice. Even though *Mmp2* was induced in *Il15ra^–/–^* mice, this increase was not statistically significant. *Mmp14* was also induced only in wildtype mice, whereas *Mmp9* was upregulated in *Il15^–/–^* mice though not in *Il15ra^–/–^* mice (**Figure 2**). Wildtype mice also showed upregulation of tissue inhibitor of MMP gene *Timp1*, whereas *Il15^–/–^* and *Il15ra^–/–^* mice did not show significant change in *Timp1* or *Timp2* expression. Overall, livers of *Il15^–/–^* and *Il15ra^–/–^* mice exposed to CCl_4_ upregulate collagen genes similarly to wildtype mice but show blunted induction of *Tgfb* and variable induction of matrix modifying enzyme genes and that the latter two may contribute to reduced liver fibrosis in IL-15 or IL-15Rα deficient mice compared to wildtype controls.

### Impaired collagen deposition and immune cell recruitment in IL-15Rα-deficient mice

**Figure 3:**
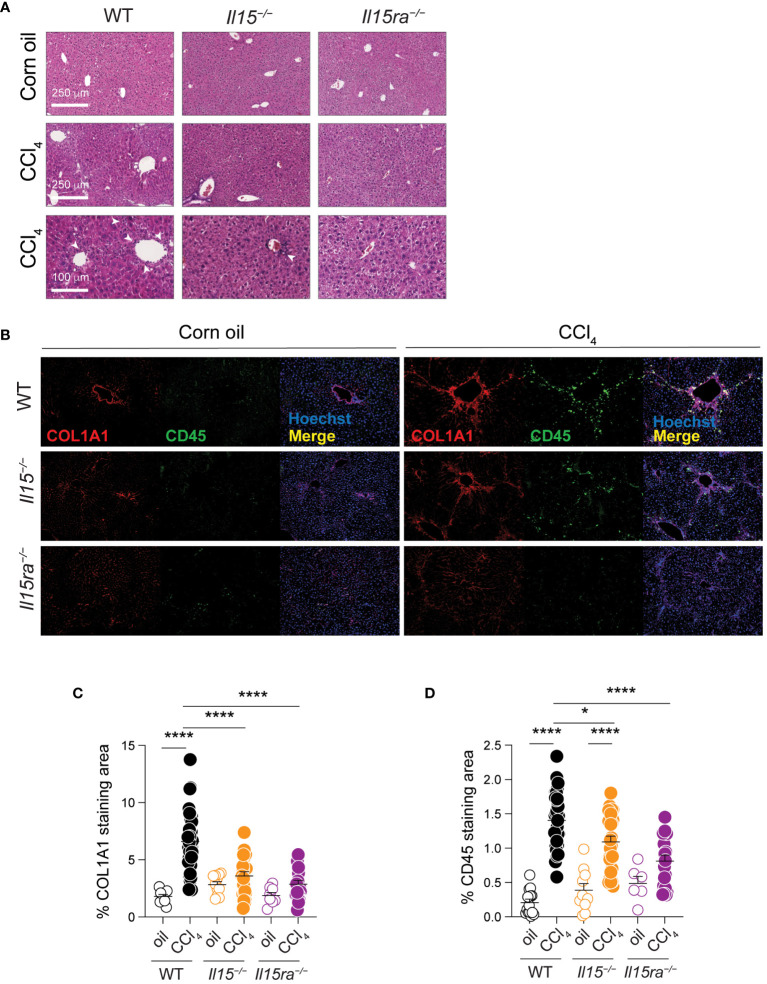
IL15 or IL15Rα deficiency reduces mononuclear cell infiltration in livers of CCl_4_-treated mice. **(A)** Hematoxylin and eosin-stained sections of livers from oil- and CCl_4_-treated WT, *Il15^–/–^* and *Il15ra^–/–^* mice. Lower (bar = 100 μm) and higher (bar = 250 μm) magnification images are shown for CCl_4_-treated to indicate mononuclear cell infiltration in the latter (white arrowheads). Data are representative of 4-5 mice per group from two independent experiments. **(B)** Representative immunofluorescence staining of liver sections of from oil- and CCl_4_-treated WT, *Il15^–/–^* and *Il15ra^–/–^* mice for COL1A1 in red and CD45 in green color. Nuclei were counter stained with Hoechst 33342. Images are representative of 2-3 mice per group from two independent experiments. Quantification of **(C)** COL1A1 staining and **(D)** CD45 infiltration areas was performed on at least five randomly selected fields from different liver sections collected from 2 to 3 mice per group. Mean + SEM are shown. One-way ANOVA with Tukey’s post-hoc test: *p<0.05, ****p<0.0001.

Examination of hematoxylin/eosin-stained liver sections revealed notable infiltration of mononuclear cells in CCl_4_-treated livers of wildtype mice in periportal areas that was markedly reduced in *Il15^–/–^* mice and further reduced in *Il15ra^–/–^* mice (**Figure 3A**). When we assessed the distribution CD45^+^ cells that labels all the leukocytes, along with collagen I (COL1A1) expression in the liver sections, the livers of oil-treated control mice showed COL1A1 localized to the portal area with only a few CD45^+^ cells. The livers of CCl_4_-treated wildtype mice showed increased COL1A1 deposition and marked infiltration of CD45^+^ cells that accumulated along the collagen fibers (**Figures 3B–D**). CCl_4_-treated *Il15^–/–^* mice showed significantly reduced COL1A1 deposition and CD45^+^ cell infiltration, confirming the profibrogenic role of IL-15, and suggesting the involvement of hepatic leukocytes in the pathogenic process. Notably, CCl_4_-treated *Il15ra^–/–^* mice showed even less staining of COL1A1 and CD45^+^ cell infiltration compared to *Il15^–/–^* mice, clearly indicating that IL-15 signaling via IL-15Rα-dependent trans-presentation promotes liver fibrosis via promoting inflammatory cell recruitment. Moreover, the differences observed between IL-15-deficient and IL-15Rα-deficient mice suggest a possible antifibrogenic role for IL-15Rα-independent IL-15 signaling.

### IL-15Rα deficiency attenuates macrophage recruitment into the liver

**Figure 4:**
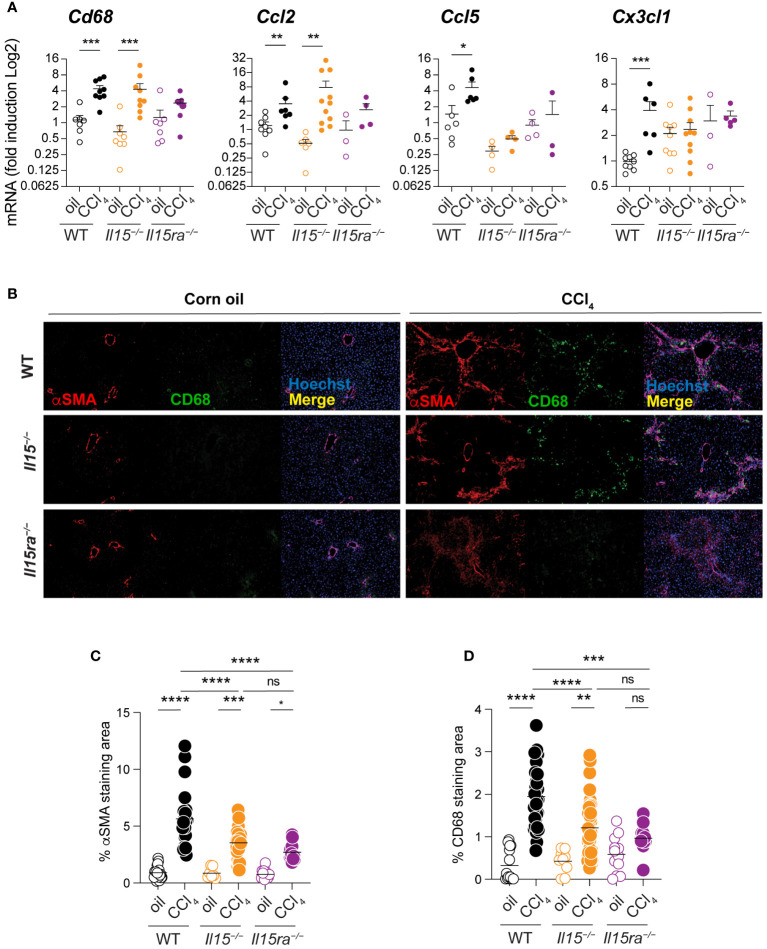
Loss of IL15 or IL15Rα reduces CD68^+^ macrophage infiltration in livers of CCl_4_-treated mice. **(A)** *Cd68*, *Ccl2*, *Ccl5* and *Cx3cl1* gene expression was assessed in the livers of oil- and CCl_4_- treated WT, *Il15^–/–^* and *Il15ra^–/–^* mice. Mean + SEM. Mann-Whitney’s test: *p<0.1; ***p<0.001; ****p<0.0001, ns, not significant. N=3-10 mice per group. **(B)** Tissues were stained with alpha-SMA and CD68 antibodies. Nuclei were counter stained with Hoechst. Images are representative of 2-3 mice per group from two independent experiments. Quantification of **(C)** αSMA staining and **(D)** CD68 infiltration areas was performed on at least five randomly selected fields from different liver sections collected from 2 to 3 mice per group. Mean + SEM. One-way ANOVA with Tukey’s post-hoc test: *p<0.05, **p<0.1, ****p<0.0001.

As myeloid cells play a key role in liver fibrosis (11) and IL-15Rα-independent IL-15 signaling impacts macrophage functions (34), we examined macrophage infiltration into the livers of CCl_4_-treated *Il15^–/–^*, *Il15ra^–/–^* and wildtype mice. CCl_4_-treated wildtype mice showed a significant increase in the transcript levels of *Cd68*, a marker for macrophages, that coincided with the upregulation of *Ccl2* chemokine gene that codes for macrophage chemotactic protein-1 (MCP-1) (**Figure 4A**). CCl_4_-treated *Il15^–/–^* mice also showed an increase in *Cd68* and *Ccl2* expression, whereas these genes were not upregulated in CCl_4_-treated *Il15ra^–/–^* mice (**Figure 4A**). CCl_4_-treated wildtype mice livers also upregulated *Ccl5* and *Cx3Cl1* genes, which encode the chemokines CCL5/RANTES and CX3CL1/Fractalkine, respectively, that are also chemotactic for monocytes and lymphocytes (**Figure 4A**). Neither *Il15^–/–^* nor *Il15ra^–/–^* mice upregulated *Ccl5* and *Cx3Cl1* genes following CCl_4_ treatment. We next examined the staining of CD68^+^ macrophages in the livers of CCl_4_-treated mice. Consistent with *Cd68* gene expression, CCl_4_-treated wildtype and *Il15^–/–^* mice showed increased numbers of CD68^+^ cell infiltration into the liver that localized to the areas of α smooth muscle cell actin (αSMA)-staining myofibroblasts (**Figure 4B**). In contrast, *Il15ra^–/–^* mice displayed markedly less αSMA staining and very few CD68^+^ cells in the liver. In the normal liver, CD68 is expressed in only a half of liver resident KCs, which are damaged during liver injury and are replenished by monocyte-derived macrophages that express CD68 (40–44). Hence, the increase in the number of CD68^+^ cells in the livers of CCl_4_ treated mice injury results mainly from infiltrating macrophages. Quantitation of the αSMA and CD68 staining areas showed significantly less staining in *Il15^–/–^* mice compared to wildtype mice that were further reduced in *Il15ra^–/–^* mice (**Figures 4C, D**). These data further confirm the requirement of IL-15 signaling via IL-15Rα-dependent trans-presentation in promoting the accumulation of macrophages during hepatic fibrogenesis.

### IL-15 signaling via IL-15Rα promotes proinflammatory monocyte recruitment during hepatic fibrogenesis

**Figure 5:**
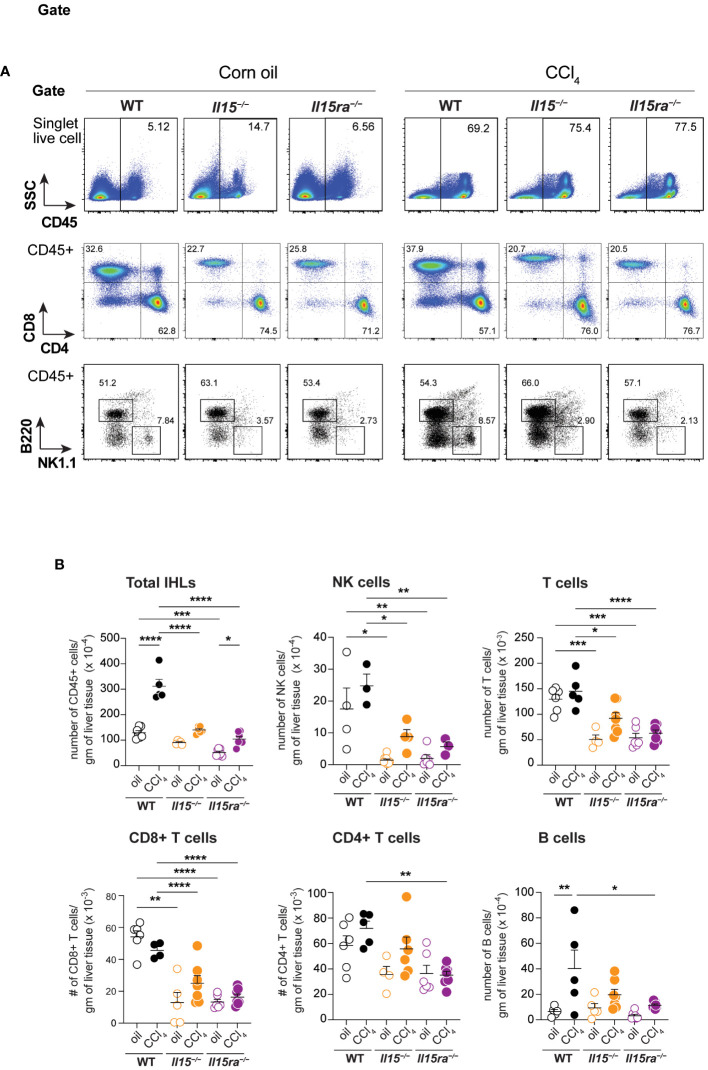
Phenotype of lymphoid cells in the livers of oil- and CCl_4_- treated mice. **(A)** Flow cytometry analyzes of representative mice from each group for the different lymphoid cell subsets in the liver are shown. **(B)** Numbers of total and the indicated IHLs were calculated from the proportion of cells determined by flowcytometry and the cell yield per gram of liver tissue. Two-way ANOVA with Tukey’s post-hoc test: *p<0.05, **p<0.01, ***p<0.001, ****p<0.0001.

To characterize the immune cells that infiltrated the liver during CCl_4_-induced liver fibrosis, we isolated intrahepatic leukocytes (IHL) and analyzed them by flow cytometry (**Figure 5A**). The proportion and total numbers of CD45^+^ IHLs was significantly higher in the CCl_4_-treated wildtype mice when compared to oil-treated controls (**Figures 5A, B**). Even though the proportion of CD45^+^ IHLs increased in CCl_4_-treated *Il15^–/–^* mice, their absolute numbers did not increase (**Figures 5A, B**). CCl_4_-treated *Il15ra^–/–^* mice did not show an increase in proportion CD45^+^ IHLs but their total numbers were significantly higher than in the corresponding oil-treated controls. The absolute number of CD45^+^ IHLs recovered in CCl_4_-treated *Il15^–/–^* and *Il15ra^–/–^* mice were comparable between them but were significantly lower than that of wildtype mice (**Figure 5B**), indicating that IL-15 signaling via IL-15Rα promotes leukocyte infiltration during hepatic fibrogenic response. As IL-15 signaling is required for the homeostasis of NK and CD8^+^ T cells (14, 15), we examined their numbers in *Il15^–/–^* and *Il15ra^–/–^* mice after fibrosis induction. The numbers of NK cells, CD3^+^ T cells and CD8^+^ T cells were not upregulated by CCl_4_ treatment, but they were significantly reduced in the livers of both vehicle-treated and CCl_4_-treated *Il15^–/–^* and *Il15ra^–/–^* mice when compared to the corresponding wildtype mice (**Figures 5A, B**). CCl_4_ treatment increased B cell numbers in the liver, but this increase was significantly reduced in *Il15ra^–/–^* mice (**Figure 5B**).

**Figure 6:**
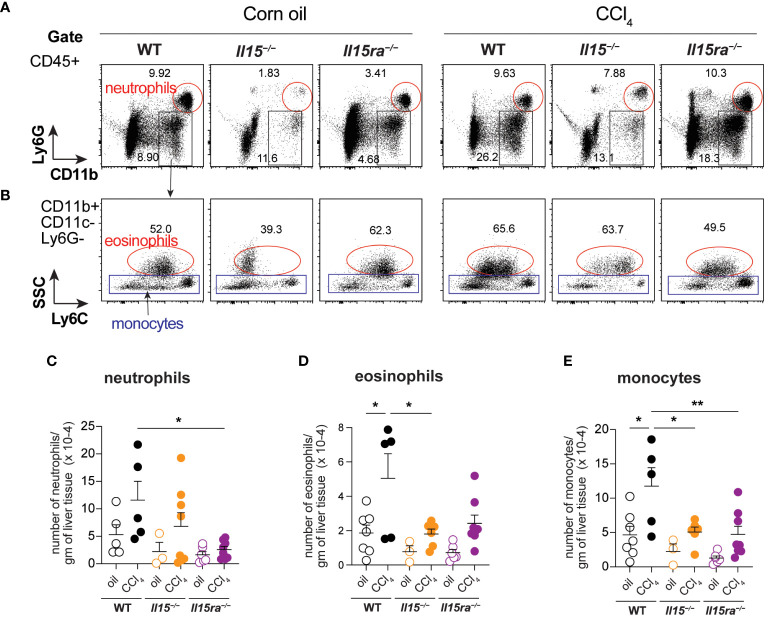
Phenotype of myeloid cells in the livers of oil- and CCI_4_- treated mice. **(A, B)** Flow cytometry analyses of representative mice from each group for the different myeloid cell subsets in the liver are shown. **(C–E)** Total numbers of neutrophils **(C)**, eosinophils **(D)** and monocytes **(E)** within the CD45^+^CD11b^+^Ly6G^-^CD11c^-^Eo^-^ gate. Representative data from at least 3 mice in the oil-treated group and 5 mice in the CCl4-treated group are shown. Mean + SEM from at least 3 mice in the oil-treated group and 5 mice in the CCl4-treated group are shown. One-way ANOVA with Tukey’s post-hoc test: * p<0.05, ** p<0.01.

**Figure 7:**
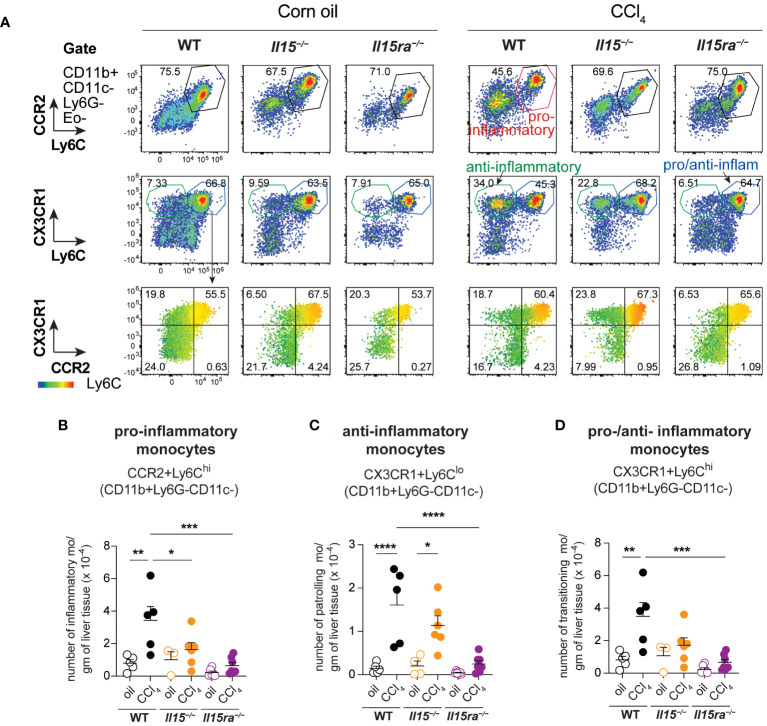
IL-15 signaling deficiency reduces the infiltration of monocytes during CCl_4_-induced liver fibrosis. **(A)** Flow cytometry analyzes of representative mice from each group for the different monocyte cell subsets in the liver are shown. **(B–D)** the numbers of monocyte subsets were calculated from the proportion of cells determined by flow cytometry and the cell yield per gram of liver tissue. As most of the Ly6C high cells are also positive for CCR2 and CX3CR1 (A, bottom panel), the same subsets are included in the pro-inflammatory monocytes **(B)** and in the pro-/anti-inflammatory monocytes **(D)**. Mean + SEM from at least 3 mice in the oil-treated group and 5 mice in the CCl_4_-treated group are shown. One-way ANOVA with Tukey’s post-hoc test: *p<0.05, **p<0.01, ***p<0.001, ****p<0.0001.

Phenotypic segregation of CD45^+^ IHL-myeloid cell subsets into neutrophils (CD11b^+^Ly6G^+^), eosinophils (CD11b^+^CD11c^–^Ly6G^–^Ly6C^Lo^) and monocytes (CD11b^+^CD11c^–^Ly6G^–^) showed comparable frequencies of these myeloid cell subsets in the three genotypes within the non-treated and CCl_4_-treated groups (**Figures 6A, B**). However, CCl_4_-treated wildtype mice showed a significant increase in the absolute numbers of neutrophils, eosinophils and CD11b^+^ monocytes (**Figures 6C–E**). The increase in these cell numbers was minimal in *Il15^–/–^* and *Il15ra^–/–^* mice, and the number of monocytes in these mice were significantly lower than in wildtype mice after CCl_4_ treatment. Even though the hepatic CD11b^+^CD11c^–^Ly6G^–^ population includes both resident KCs and monocyte-derived macrophages, the increase in their numbers during CCl_4_-induced liver fibrosis results mainly from infiltrating monocyte derived macrophages (11, 41). Next, we segregated CD11b^+^CD11c^–^Ly6G^–^ macrophages into inflammatory (Ly6C^Hi^CCR2^+^) and anti-inflammatory (Ly6C^Lo^CCR2^-^CX3CR1^+^) subsets (**Figure 7A**). In wildtype mice, the proportion of the inflammatory subset appear decreased following CCl_4_ treatment, and the anti-inflammatory subset increased, although total numbers for both subsets showed a net increase (**Figures 7A–C**). Absolute numbers of pro-inflammatory monocytes did not increase in either *Il15^–/–^* or *Il15ra^–/–^* mice following CCl_4_ treatment compared to vehicle-treated controls, whereas anti-inflammatory monocytes were increased in CCl_4_-treated *Il15^–/–^* mice but not *Il15ra^–/–^* mice (**Figures 7B, C**). Most of the CCR2^+^ monocytes were also positive for CX3CR1 and expressed high levels of Ly6C (**Figure 7A**, last row and **Figure 7D** **;** **Supplementary Figure S1**). These Ly6C^hi^CCR2^+^CX3CR1^+^ cells may represent pro-inflammatory cells transitioning to anti-inflammatory cells (11, 13). The number of these transitional cells increased in CCl_4_-treated wildtype mice, but not in *Il15^–/–^* or *Il15ra^–/–^* mice (**Figure 7D**). Ly6C^Hi^ monocytes expressed invariably both CCR2 and CX3CR1, whereas Ly6C^Lo^ monocytes expressed variable levels of CCR2 and CX3CR1 (**Supplementary Figure S1**). Collectively, these data indicate that IL-15 signaling via IL-15Rα promotes monocyte recruitment and their differentiation towards proinflammatory phenotype during hepatic fibrogenesis.

## Discussion

Liver fibrosis results from chronic inflammatory response and the accompanying profibrogenic pathways. Proinflammatory cytokines play a key role in the pathogenesis of liver fibrosis. IL-15 is a pro-inflammatory cytokine induced by myriad of inflammatory stimuli (17–20). In this study, we show that IL-15 promotes pathogenesis of liver fibrosis through IL-15Rα-dependent pro-inflammatory macrophage recruitment.

IL-15 is a pleiotropic cytokine, with major impacts on lymphoid cells. The homeostasis of NK cells, memory CD8^+^ T cells, NKT cells, γδT cells, intraepithelial cells and is dependent on IL-15 and its trans-presented by IL-15Rα, as mice lacking either IL-15 or IL-15Rα harbor diminished numbers of these cells (14, 15, 45, 46). Compared to peripheral lymphoid organs, the liver harbors a greater proportion of innate immune cells of lymphoid and myeloid origin (47–49). Human liver contains higher proportion of NK cells, while NKT cells are present in greater proportions in murine livers (50). The role of intrahepatic NK and NK T cells in liver fibrosis is complex with certain subsets conferring protection while others promote fibrosis progression. Various studies have established a pathogenic role for NKT cells in the pathogenesis of liver fibrosis in humans and in pre-clinical models (6, 47, 51–54). In contrast to NKT cells, NK cells and CD8^+^ T cells have been shown to prevent the progression of liver fibrosis by killing activated hepatic stellate cells that are the major producers of collagen and extra-cellular matrix (28, 55–57). Consistent with the earlier reports on *Il15^–/–^* and *Il15ra^–/–^* mice, and CCl_4_-treated *Il15ra^–/–^* mice, we observed in the current study reduced numbers of NK cells in vehicle-treated and CCl_4_-treated *Il15^–/–^* and *Il15ra^–/–^* mice. However, the paucity of NK cells did not result in increased liver fibrosis CCl_4_-treated *Il15^–/–^* and *Il15ra^–/–^* mice in our study.

Even though the loss of IL-15 or IL-15Rα diminished CCl_4_-induced liver fibrosis, this reduction was more pronounced in *Il15ra^–/–^* mice than in *Il15^–/–^* mice. This trend was also observed for the number of total IHLs, neutrophils and proinflammatory and anti-inflammatory monocytes infiltrating the liver. Moreover, the expression of *Cd68* and *Ccl2* genes was even comparable between *Il15^–/–^* and wildtype mice but reduced in *Il15ra^–/–^* mice. Even though these differences were not significant between *Il15^–/–^* and *Il15ra^–/–^* mice, they are intriguing because if IL-15 were to exert only pro-inflammatory effect, the loss of IL-15 should result in more pronounced reduction in fibrosis and the associated molecular changes than those caused by the loss of IL-15Rα. These findings suggest that in the absence of IL-15Rα, IL-15 signaling via IL-15Rβγ may exert an anti-fibrotic effect, resulting in less fibrosis in *Il15ra^–/–^* mice than in *Il15^–/–^* mice. Nonetheless, such anti-fibrotic function of IL-15 signaling via IL-15Rβγ is clearly eclipsed by IL-15Rα-mediated proinflammatory signaling.

The increased fibrosis in CCl_4_-treated *Il15ra^–/–^* mice reported in an earlier study (32) stands in stark contrast to our fining of reduced liver fibrosis in our study. Reasons for this discrepancy are not completely clear. One potential source of this difference could be the difference in the sex of mice used. Whereas Jiao et al., used female mice (32), we routinely use male mice for liver fibrosis studies to overcome the confounding influence of sex hormones on inflammatory cytokine production in the liver (37). Hence, we tested liver fibrosis development in female *Il15^–/–^* and *Il15ra^–/–^* mice following CCl_4_ treatment. Similarly, to male mice (**Figures 1C–E**), female *Il15^–/–^* and *Il15ra^–/–^* mice also developed reduced liver fibrosis in our study (**Supplementary Figure S2**), indicating that sex is an unlikely factor in the observed discrepancy with the findings of Jiao et al. (37). A second source of discrepancy could be the different genetic background of the mice used. Whereas Jiao et al. used *Il15ra^–/–^* mice were in B6/129 mixed background (32, 37), *Il15^–/–^* and *Il15ra^–/–^* mice used in our study have been backcrossed to the C57BL/6 background. However, as we observed similarly reduced liver fibrosis both *Il15^–/–^* and *Il15ra^–/–^* mice under the same experimental settings, our findings strongly support the profibrogenic role of IL-15 signaling in hepatic fibrogenesis. Jiao et al., attributed the increased liver fibrosis to the direct inhibitory effect of IL-15Rα mediated-signaling on the fibrogenic response of HSCs (37). Our findings indicate that any such antifibrogenic activities of IL-15Rα-dependent IL-15-signaling are insufficient to counter the profibrogenic effects of IL-15.

Another notable observation is the induction of collagen genes *Col1a1* and *Col1a3* in *Il15^–/–^* and *Il15ra^–/–^* mice to the level comparable to that of wildtype mice, although the amount of collagen deposition as revealed by Sirius red or Masson’s trichrome staining was significantly reduced in the IL-15 or IL-15Rα deficient mice. Myofibroblasts are the primary source of collagens, and αSMA labeling showed marked reduction in *Il15^–/–^* and *Il15ra^–/–^* mice, in line with the reduced induction of *Tgfb* gene, the key driver of myofibroblast differentiation of HSCs. The reasons for reduced collagen production despite elevated gene induction *Il15^–/–^* and *Il15ra^–/–^* mice remain unclear. Given that IL-15 signaling can activate mTOR signaling (58), further investigations are needed to test the possibility that IL-15Rα-mediated profibrogenic IL-15 signaling may be involved in translation of collagen gene transcripts in myofibroblasts.

MMPs are key mediators of tissue repair and reorganization, and their deregulated expression and modulation by TIMPs is a characteristic of fibrotic diseases. Among the many MMPs, we examined the expression of three candidates MMP2, MMP9 and MMP-14. MMP2 is increased during fibrosis and exerts anti-fibrotic effect, whereas MMP14 is upregulated during fibrosis resolution (39, 59). MMP2 and MMP14 are upregulated in CCL_4_-treated control mice, their expression was not increased in both *Il15^–/–^* and *Il15ra^–/–^* mice, reflecting the pro-fibrogenic role of IL-15 signaling. Notably, MMP9, which promotes apoptosis of HSCs (60), was elevated only in *Il15^–/–^* mice but not in control or and *Il15ra^–/–^* mice, suggesting that one of the mechanisms by which IL-15 signaling promotes fibrosis could occur by preventing apoptosis of HSCs, for which IL-15Rα may be dispensable. These possibilities remain to be tested.

Liver tissue resident KCs and infiltrating monocyte-derived macrophages play key roles in hepatic fibrogenic response (41). KCs, which originate from yolk sac-derived progenitors and are key mediators of liver homeostasis, have limited self-renewal capacity, and thus are replenished by monocyte-derived macrophages that acquire KC properties (44). Thus, the increase in the number of CD68^+^ macrophages in the liver of CCl_4_-treated mice results mainly from infiltrating monocyte-derived macrophages. The infiltrating macrophages display changes in chemokine receptor expression that correlate with their functional differentiation (11). Strikingly, loss of IL-15 or IL-15Rα during CCl_4_- induced liver fibrosis resulted in significant reduction in the number of CCR2^+^Ly6C^Hi^ proinflammatory macrophages compared to wildtype mice, whereas the number of CX3CR1^+^Ly6C^Lo^ anti-inflammatory macrophages were significantly reduced only in *Il15ra^–/–^* mice compared to wildtype controls. During hepatic fibrogenic response, the inflammatory macrophages differentiate towards restorative macrophages that promote fibrosis resolution (61). However, the reduced number of anti-inflammatory macrophages in *Il15ra^–/–^* mice is unlikely to result from a block in the differentiation of pro-inflammatory monocytes, as the recruitment of the latter itself is severely impaired in the absence of IL-15Rα.

In conclusion, our findings show that IL-15 signaling via IL-15Rα is strongly pro-inflammatory and profibrogenic during liver fibrosis. IL-15 has been well-established as an important cytokine for immune cell homeostasis that also plays pathogenic roles in autoimmune diseases (18). Hence, intense efforts are being made in developing biomolecules that can inhibit the proinflammatory effects of IL-15 signaling to prevent disease progression and facilitate recovery without impairing its homeostatic functions (62). Such antagonists of IL-15 signaling include soluble IL-15Rα (63), anti-IL-15Rβ antibodies (64, 65), anti-IL-15 antibodies (66–69), mutant IL-15 (70) and small molecule inhibitors (71). Notably, a mutant IL-15 construct with impaired ability to bind IL-2Rβ (NANTIL-15) selectively blocked IL-15 signaling via the trimeric IL-15Rα/IL-2Rβ/γc receptor and reduced inflammation in a collagen-induced arthritis model, without inhibiting NK or CD8+ T cell homeostasis (70). The possibility of targeting IL-15 signaling in liver fibrosis remains to be tested, and should take into consideration that IL-15 treatment has been shown to be beneficial in kidney and pancreatic fibrosis models, possibly via reducing TGFβ production and its profibrotic functions by unknown mechanisms (72–75). In the light of these reports, our findings raise the possibility that the pro-inflammatory role of IL-15 is crucial for initiation and progression of inflammatory diseases, whereas the potential impact of IL-15 signaling on TGFβ pathway may reduce disease severity. Testing IL-15 signaling agonists and antagonists in the same model at different stages of disease would be crucial to determine which approach would be more effective in treating inflammatory diseases.

## Data availability statement

The raw data supporting the conclusions of this article will be made available by the authors, without undue reservation.

## Ethics statement

The animal study was approved by Université de Sherbrooke, Faculty of Medicine and Health Sciences Animals Ethics committee (2020-2732). The study was conducted in accordance with the local legislation and institutional requirements.

## Author contributions

SR: Writing – review & editing, Writing – original draft, Validation, Supervision, Resources, Project administration, Methodology, Investigation, Funding acquisition, Formal analysis, Conceptualization. MC: Writing – review & editing, Writing – original draft, Validation, Methodology, Investigation, Formal analysis, Conceptualization. BV: Writing – review & editing, Methodology, Investigation, Conceptualization. SA: Writing – review & editing, Methodology, Investigation, Formal analysis. FR: Writing – review & editing, Methodology, Investigation, Formal analysis. SI: Project administration, Writing – review & editing, Writing – original draft, Supervision, Resources, Methodology, Investigation, Funding acquisition, Conceptualization.
