# Supplementary material for: Profibrogenic role of IL-15 through IL-15 receptor alpha-mediated trans-presentation in the carbon tetrachloride-induced liver fibrosis model
Source: Front Immunol. 2024 Jun 11;15:1404891. doi: 10.3389/fimmu.2024.1404891 (PMC11196400; doi:10.3389/fimmu.2024.1404891)
Supplement: Supplementary file 1 [file DataSheet_1.docx]

**Profibrogenic role of IL-15 through IL-15 receptor alpha-mediated trans-presentation in the carbon tetrachloride-induced liver fibrosis model**

**Maryse Cloutier*, Bhavesh Variya*, et al.,**

**Supplementary Table S1.** Antibodies used for immunohistochemistry or immunofluorescence.

| **Antibody** | **Supplier** | **Clone** | **Cat. #** |
| --- | --- | --- | --- |
| Mouse anti-αSMA | Abcam | 1A4 | ab7817 |
| Rabbit anti-CD68 | Abcam | Polyclonal | ab125212 |
| Rabbit anti-COL1A1 | Abcam | Polyclonal | ab34710 |
| Rat anti-CD45  Alexa Fluor® 488 | Biolegend | 30-F11 | 103122 |
| Goat anti-rabbit Alexa Fluor™ 488 | Thermo Fisher | Polyclonal | A-11034 |
| Goat anti-rabbit Alexa Fluor™ 568 | Thermo Fisher | Polyclonal | A-11036 |
| Goat anti-mouse  Alexa Fluor™ 568 | Thermo Fisher | Polyclonal | A-11031 |

**Supplementary Table S2.** List of RT-qPCR primers.

| Gene name | Gene ID | Sense primer | Anti-sense primer | Amplicon Size (bp) |
| --- | --- | --- | --- | --- |
| *Col1a1* | NM_007742.4 | CTCCCAGAACATCACCTATCAC | ACTGTCTTGCCCCAAGTTCCG | 192 |
| *Col3a1* | NM_009930.2 | AAGTCAAGGAGAAAGTGGTCG | CAGTCTCCCCATTCTTTCCAG | 179 |
| *Timp1*  *Timp2*  *Mmp2*  *Mmp9*  *Mmp14*  *Tgfb1*  *Cd68*  *Ccl2*  *Ccl5*  *Cx3cl1*  *Il15*  *36B4 (Rplp0)* | NM_011593.2  NM_011594.3  NM_008610.3  NM_013599.5  NM_008608.4  NM_011577.2  NM_001291058.1  NM_011333.3  NM_013653.3  NM_009142.3  NM_008357.3  NM_007475.5 | TTGCATCTCTGGCATCTGG  CAGGAAAGGCAGAAGGAGATG  CACCTACACCAAGAACTTCCG  AACTCACACGACATCTTCCAG  GGATGGACACAGAGAACTTCG  ATACGCCTGAGTGGCTGTCT  GTGTCTGATCTTGCTAGGACC  CATCCACGTGTTGGCTCA  TGCAGAGGACTCTGAGACAGC  TCTTCCATTTGTGTACTCTGCT  CCTTAAGAACACAGAAACCCATG  TCTGGAGGGTGTCCGCAAC | TGGTCTCGTTGATTTCTGGG  GATCATGGGACAGCGAGTG  GTCACTGTCCGCCAAATAAAC  CCACCTTGTTCACCTCATTTTG​  AACCATCGCTCCTTGAAGAC  CTGATCCCGTTGATTTCCA  TGTGCTTTCTGTGGCTGTAG  GATCATCTTGCTGGTGAATGAGT  GAGTGGTGTCCGAGCCATA  GGACTCCTGGTTTAGCTGATAG  AGGAAACACAAGTAGCACGAG  CTTGACCTTTTCAGTAAGTGG | 156  149  225  97  235  148  118  76  149  128  199  154 |

**Supplementary Table S3.** Antibodies used for Flow cytometry:

A) Myeloid cell panel

| **Antibody** | **Fluorochrome** | **Source** | **Clone** | **#Cat.** |
| --- | --- | --- | --- | --- |
| Fixable Viability Dye | e-fluor 780 | ebiosciences |  | 65-0865 |
| CD45 | Brilliant Violet 510 | Biolegend | 30-F11 | 103138 |
| CD11b | eFluor450 | ebiosciences | M1/70 | 48-011282 |
| Ly6G | PerCP | Biolegend | 1A8 | 127654 |
| Ly6C | FITC | BD | AL21 | 553104 |
| CD11c | Alexa Fluor 700 | ebiosciences | N418 | 56-0114-82 |
| CCR2 | PE | Biolegend | SA203G11 | 150610 |
| CX3CR1 | APC | Biolegend | SA011F11 | 149008 |
| MHC-II | PECy7 | Biolegend | M5/114.15.2 | 107630 |
| F4/80 | Brilliant Violet 605 | BV605 | BM8 | 123133 |

B) Lymphoid cell panel

| **Antibody** | **Fluorochrome** | **Source** | **Clone** | **#Cat.** |
| --- | --- | --- | --- | --- |
| CD45 | Brilliant Violet 605 | Biolegend | 30-F11 | 103140 |
| CD3ε | Brilliant Violet 510 | Biolegend | 145-2C11 | 100353 |
| TCRβ | PE/Dazzle594 | Biolegend | H57-597 | 109240 |
| CD4 | Alexa Fluor 700 | ebiosciences | GK1.5 | 5016851 |
| CD8α | eFluor450 | ebiosciences | 53-6.7 | 48-0081-82 |
| CD62L | APC | ImmunoTools | MEL-14 | 22159626 |
| CD44 | FITC | Biolegend | IM7 | 103006 |
| CD69 | PE-Cy7 | Biolegend | H1.2F3 | 25-0691-82 |
| NK1.1 | APC-Cy7 | Biolegend | PK136 | 108724 |


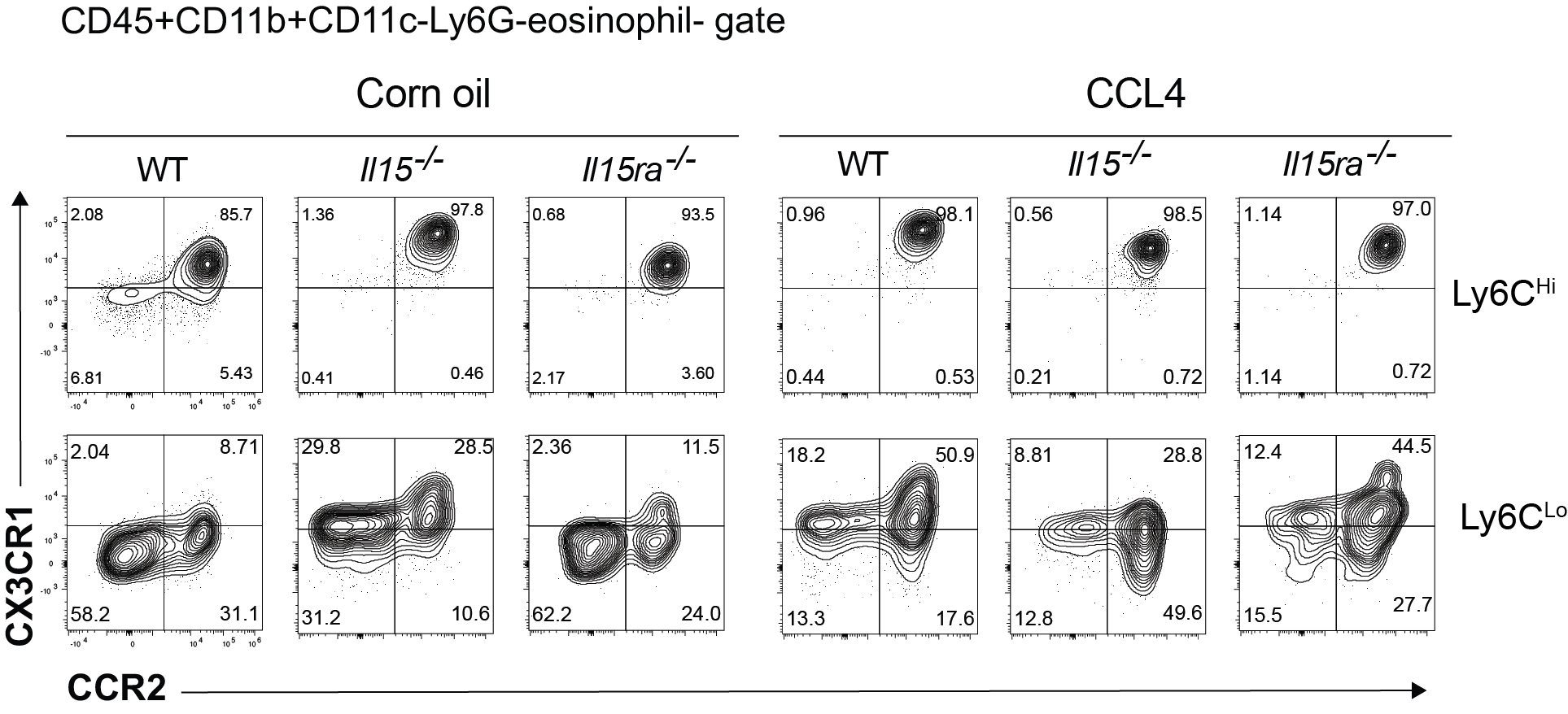


**Supplementary Figure S1**:  **Phenotype of Ly6C^Hi^  and Ly6C^Lo^ monocytes** **in the liver of control and CCL4 treated mice.** Flow cytometry analyses of CCR2 and CX3CR1 expression within the Ly6C^Hi^ and Ly6C^Lo^ monocyte subsets in livers of the indicated genotypes of mice. Representative data from at least 3 mice in each group are shown.

**Supplementary Figure S2**:  **Loss of IL-15 or IL-15Rα reduces fibrosis in the livers of CCl_4_-treated female mice.** (A) Sirius Red/fast green staining of WT, *Il15^–/–^* and *Il15ra^–/–^* liver tissues from oil- and CCl_4_- treated female mice. Representative data from at least 3-5 mice in each group are shown. (B) Quantification of Sirius Red stained area from seven random fields of the liver section from 3 to 4 mice per group. Mean + SEM. Two-way ANOVA with Tukey’s post-hoc test: * p<0.05; **; p<0. 01 ***; p<0.001; **** p<0.0001.
